# Sex Disparities and Neutralizing-Antibody Durability to SARS-CoV-2 Infection in Convalescent Individuals

**DOI:** 10.1128/mSphere.00275-21

**Published:** 2021-08-25

**Authors:** Alena J. Markmann, Natasa Giallourou, D. Ryan Bhowmik, Yixuan J. Hou, Aaron Lerner, David R. Martinez, Lakshmanane Premkumar, Heather Root, David van Duin, Sonia Napravnik, Stephen D. Graham, Quique Guerra, Rajendra Raut, Christos J. Petropoulos, Terri Wrin, Caleb Cornaby, John Schmitz, JoAnn Kuruc, Susan Weiss, Yara Park, Ralph Baric, Aravinda M. de Silva, David M. Margolis, Luther A. Bartelt

**Affiliations:** a Department of Medicine, Division of Infectious Diseases, University of North Carolina School of Medicine, Chapel Hill, North Carolina, USA; b Centre of Excellence in Biobanking and Biomedical Research, Molecular Medicine Research Center, University of Cyprus, Nicosia, Cyprus; c Department of Microbiology and Immunology, University of North Carolina School of Medicine, Chapel Hill, North Carolina, USA; d Department of Epidemiology, University of North Carolina at Chapel Hill, Chapel Hill, North Carolina, USA; e Department of Medicine, University of North Carolina School of Medicine, Chapel Hill, North Carolina, USA; f LabCorp-Monogram Biosciences, South San Francisco, California, USA; g Department of Pathology & Laboratory Medicine, University of North Carolina School of Medicine, Chapel Hill, North Carolina, USA; h UNC HIV Cure Center, University of North Carolina School of Medicine, Chapel Hill, North Carolina, USA; University of Maryland School of Medicine

**Keywords:** antibodies, SARS-CoV-2, immunology, neutralizing antibodies, neutralizing

## Abstract

The coronavirus disease 2019 (COVID-19) pandemic, caused by severe acute respiratory syndrome-related coronavirus 2 (SARS-CoV-2) has now caused over 2 million deaths worldwide and continues to expand. Currently, much is unknown about functionally neutralizing human antibody responses and durability to SARS-CoV-2 months after infection or the reason for the discrepancy in COVID-19 disease and sex. Using convalescent-phase sera collected from 101 COVID-19-recovered individuals 21 to 212 days after symptom onset with 48 additional longitudinal samples, we measured functionality and durability of serum antibodies. We also evaluated associations of individual demographic and clinical parameters with functional neutralizing antibody responses to COVID-19. We found robust antibody durability out to 6 months, as well as significant positive associations with the magnitude of the neutralizing antibody response and male sex and in individuals with cardiometabolic comorbidities.

**IMPORTANCE** In this study, we found that neutralizing antibody responses in COVID-19-convalescent individuals vary in magnitude but are durable and correlate well with receptor binding domain (RBD) Ig binding antibody levels compared to other SARS-CoV-2 antigen responses. In our cohort, higher neutralizing antibody titers are independently and significantly associated with male sex compared to female sex. We also show for the first time that higher convalescent antibody titers in male donors are associated with increased age and symptom grade. Furthermore, cardiometabolic comorbidities are associated with higher antibody titers independently of sex. Here, we present an in-depth evaluation of serologic, demographic, and clinical correlates of functional antibody responses and durability to SARS-CoV-2 which supports the growing literature on sex discrepancies regarding COVID-19 disease morbidity and mortality, as well as functional neutralizing antibody responses to SARS-CoV-2.

## INTRODUCTION

Over 12 months have passed since the emergence and eventual global spread of the novel coronavirus, SARS-CoV-2, the agent of the COVID-19 pandemic. As SARS-CoV-2 continues to spread and mutate across naive and previously exposed populations, increased understanding of the breadth and durability of individual humoral responses to natural infection is needed to assess the reinfection risk of individuals and also to guide the deployment of and to inform recently authorized vaccines and antibody-based therapies. Recent work has shown that SARS-CoV-2 can stimulate the production of highly neutralizing antibodies directed against the spike protein (S) which is necessary for viral attachment, fusion, and entry into host cells (1, 2). We and others have shown that antibodies directed against the angiotensin-converting enzyme 2 (ACE2) receptor binding domain (RBD) of the S protein consistently demonstrate a strong correlation with functional neutralization (3–5) and are protective in nonhuman primate and rodent models (6–9). Furthermore, low conservation between the RBD of SARS-CoV-2 and other non-SARS human betacoronaviruses makes RBD an appealing target for highly specific COVID-19 responses.

Serum antibody responses to endemic betacoronaviruses initially wane weeks to months after infection but remain detectable up to at least 1 year (10, 11). After SARS-CoV-1 and Middle East respiratory syndrome (MERS) CoV infections, IgG levels peak at 4 months and then slowly wane but remain detectable for at least 2 years and up to 17 years (11, 12). Although antibody seroconversion to primary SARS-CoV-2 infection is nearly universal within the first 2 weeks after symptom onset (4, 13–15), the magnitude of this response varies with symptom severity (4, 16, 17). Longevity of serum antibodies to SARS-CoV-2 S protein after vaccination as well as natural infection has been studied out to 3 months, during which time IgG, IgM, and IgA levels to most SARS-CoV-2 antigens peak and begin to decline (16, 18–20), as plasmablast and short-lived plasma cell responses wane. More recent data suggest that S protein IgG levels begin to reach a steady level with much lower rates of decline after 90 days postinfection, which lasts out to at least 8 months (5, 21, 22). Thus, studies show that SARS-CoV-2 virus-neutralizing antibodies in recovered individuals are so far durable, but the protective titer of these antibodies is unknown.

The clinical and demographic determinants of the breadth and durability of functionally neutralizing antibodies have not been studied in depth out to 6 months after SARS-CoV-2 infection. A recent study found higher ratios of RBD antibodies to nucleocapsid (N) antibodies in outpatient compared to inpatient populations (4), and some studies have suggested that there is a faster decline in S antibody levels in asymptomatic than in symptomatic individuals (4, 17). In this study, we add to growing evidence of sex disparities in neutralizing antibody responses previously seen up to 114 days post-symptom onset in predominantly urban areas (23–28). Our data support these findings out to 6 months postinfection in a previously uncharacterized cohort in a semiurban and rural population in North Carolina. Identifying these differences is critical to understanding long-term protection from natural infection as well as vaccine-induced immunity. In this study, we use both novel and established assays to characterize the binding and longevity of serum antibodies to SARS-CoV-2 RBD, spike protein N-terminal domain (NTD), and N antigens and to measure the level and durability of SARS-CoV-2-neutralizing antibodies. We further define demographic and clinical correlates of the magnitude and durability of both binding and functional antibody responses to SARS-CoV-2.

## RESULTS

### Donor characteristics.

Between 11 April and 22 July 2020, a total of 101 eligible COVID-19 convalescent plasma (CP) donors were enrolled in this study. The majority of donors donated once; however, 31 donors provided sequential donations amounting in an additional 48 serum samples. Donors were over 18 years of age, 51% male and 49% female (based on sex assigned at birth). The median age was 43 years (interquartile range 29, full range 18 to 79), which is similar to other CP donor cohorts (22, 23), and the majority identified as non-Hispanic, white/Caucasian. When stratifying by sex, no statistically significant differences were observed in median age (males, 44 years old [y/o]; females, 41 y/o), median time from symptom onset or diagnosis (males, 57 days; females, 58.5 days), and median symptom grade (males, 2; females, 2) between males and females. Donors were diagnosed with SARS-CoV-2 by either SARS-CoV-2 reverse transcriptase PCR (RT-PCR) (*n *= 79) or blood antibody testing by Emergency Use Authorization (EUA)-approved commercial assays (*n *= 22) (Table 1; see also Table S1 in the supplemental material). Donors diagnosed by antibody test either had RT-PCR-confirmed household contacts or COVID-19 symptoms without RT-PCR testing or were unable to provide a copy of their RT-PCR result. The median time from symptom onset or RT-PCR diagnosis to first donation was 57 days (full range, 21 to 121). Thirty-four donors reported comorbid conditions, the most common being hay fever and high blood pressure (Table S2). Eight donors were asymptomatic, and 93 reported symptoms. The median time of symptom duration for symptomatic donors without ongoing symptoms (72/90) was 16 days (full range, 2 to 107). Fifty-seven donors had mild-to-moderate disease (grade 1 to 2; outpatient), 14 donors had severe disease (grade 3 to 4; hospitalized), and 22 donors had unknown disease severity (Table 1). The most common symptoms reported were fatigue (89%), headache (77%), fever (74%), and muscle aches (73%) (Table S2). The majority of donors resided in central North Carolina, with the highest proportion from Orange and Wake counties (Fig. S1).

**TABLE 1:** Convalescent-phase plasma donor characteristics at time of donation^*a*^

Characteristic	No.
Age (yr)	
18–39	39
40–64	53
65–79	9
80+	0

Sex	
M	52
F	49

Parity (*n* = 48)	
Parous	26
Nulliparous	22
	
Comorbid conditions	
None	64
One	18
Two or more	16
Unknown	3
	
Race (*n* = 98)	
White/Caucasian	75
Black/African American	7
Asian	5
Pacific Islander	1
Other	10
	
Ethnicity (*n* = 98)	
Hispanic	15
Non-Hispanic	82
Unknown	1
	
ABO (*n* = 99)	
A^+^	36
A^−^	7
B^+^	7
B^−^	1
AB^+^	6
AB^−^	0
O^+^	36
O^−^	6
	
COVID-19 disease characteristics	
RT-PCR diagnosed	79
Antibody diagnosed	22
Diagnostic test unknown	1
Symptomatic	93
Asymptomatic	8
Overall symptom grade (*n* = 71)	
1 (mild)	24
2 (moderate)	33
3 (severe)	11
4 (potentially life-threatening)	3
Supplemental oxygen required (*n* = 71)	6
	
Median time	Days, range
From symptom onset or RT-PCR diagnosis to donation (*n* = 95)	57, 21–121
Of symptom duration (*n* = 70)	16, 2–107

a Plasma donor demographic and COVID-19 disease characteristics. *n* = 101 unless otherwise specified. RT-PCR, reverse transcriptase PCR. Symptom grades: grade 1 (mild), mild symptoms causing no or minimal interference with usual social and functional activities with intervention not indicated; grade 2 (moderate), moderate symptoms causing greater than minimal interference with usual social and functional activities with intervention indicated; grade 3 (severe), severe symptoms causing inability to perform usual social and functional activities with intervention or hospitalization indicated; oxygen administered via nasal cannula; grade 4 (potentially life-threatening), potentially life-threatening symptoms causing inability to perform basic self-care functions with intervention indicated to prevent permanent impairment, persistent disability, or death; hospitalization requiring intubation or use of supplemental oxygen (continuous positive airway pressure [CPAP] or oxygen administered via mask).

10.1128/mSphere.00275-21.1 FIG S1 Map of donor zip codes. A county map was generated using zip code data of donors who were located in North Carolina (*n* = 86); home site for study is indicated by yellow star. Download FIG S1, TIF file, 8.6 MB. Copyright © 2021 Markmann et al. 2021 Markmann et al. https://creativecommons.org/licenses/by/4.0/ This content is distributed under the terms of the Creative Commons Attribution 4.0 International license.

10.1128/mSphere.00275-21.8 TABLE S1 Donor SARS-CoV-2 diagnostic and symptom data. Site 1 indicates home site for this study. Labs 1 and 2 are testing labs located within North Carolina that accounted for at least five samples. “Other site” and “other lab” represent other U.S. hospitals or laboratories. RT-PCR, reverse transcriptase PCR; Ab, antibody; *, required mechanical ventilation; Ϯ, could not provide RT-PCR test documentation. Download Table S1, DOCX file, 0.03 MB. Copyright © 2021 Markmann et al. 2021 Markmann et al. https://creativecommons.org/licenses/by/4.0/ This content is distributed under the terms of the Creative Commons Attribution 4.0 International license.

10.1128/mSphere.00275-21.9 TABLE S2 Donor comorbidity and COVID-19 symptom breakdown. (Top) Comorbid conditions reported by 34 donors. (Bottom) “Other symptoms” include abdominal pain (1), abdominal bloating (2), weight loss (1), leg pain, worsening vision (2), pruritic scalp (1), general symptom recurrence (1), seizures (1), excess thirst (1), urinary symptoms (1), syncopal episode (1), and severe nightmares (1). Numbers in parentheses = *n* participants. Download Table S2, DOCX file, 0.02 MB. Copyright © 2021 Markmann et al. 2021 Markmann et al. https://creativecommons.org/licenses/by/4.0/ This content is distributed under the terms of the Creative Commons Attribution 4.0 International license.

### Neutralization and binding antibody assays.

**FIG 1:**
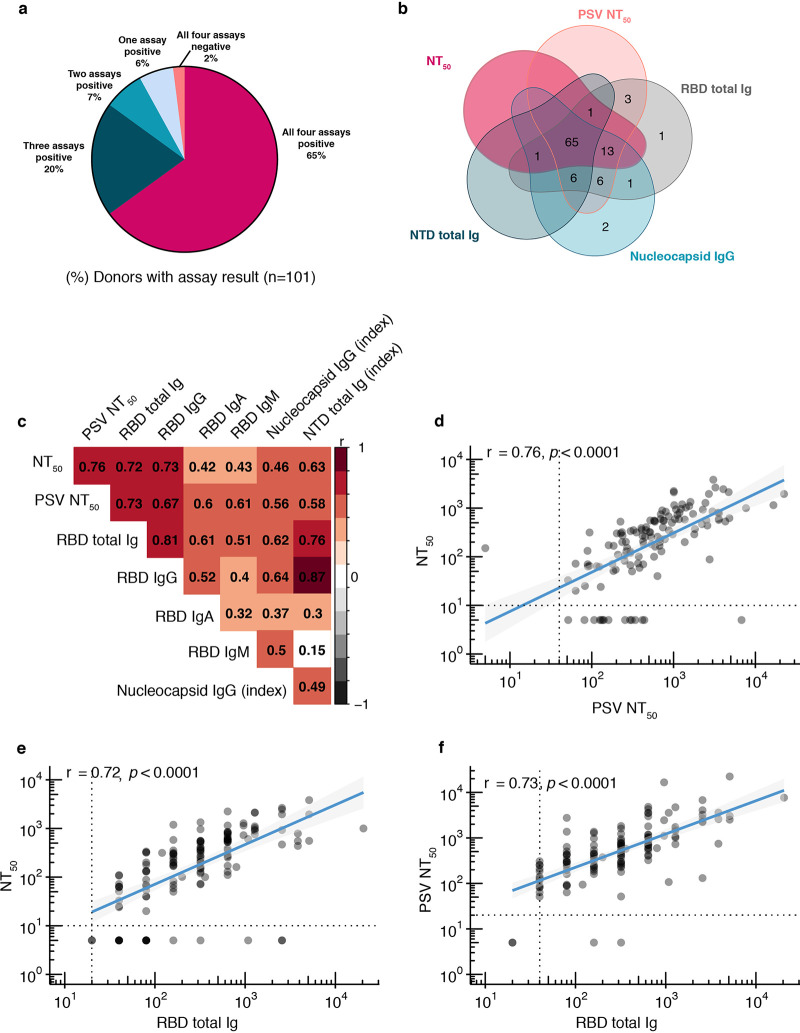
Neutralizing and binding antibody results. (a) Pie chart with overall assay results for all 101 donors, four assays shown (reporter virus neutralization assay, RBD and NTD total Ig assays, and nucleocapsid IgG assay). (b) Venn diagram showing overlap among five assays (reporter virus neutralization assay, pseudovirus neutralization assay, RBD and NTD total Ig assays, and nucleocapsid IgG assay). (c) Heat map of Spearman’s correlation coefficients examining the association between all assays performed. Red represents positive association between assays, and black represents negative associations. Nonsignificant correlation coefficients (*P* > 0.05) are left blank. (d) Reporter virus NT_50_ dilution plotted against pseudovirus NT_50_ dilution, *P* < 0.0001. (e) Reporter virus NT_50_ dilution plotted against RBD total Ig antibody level (endpoint titer), *P* < 0.0001. (f) Pseudovirus NT_50_ dilution plotted against RBD total Ig antibody level (endpoint titer), *P* < 0.0001. For panels d to f, nonparametric, two-tailed Spearman’s rank correlation was used to calculate correlation coefficients (*r*) and *P* values (*p*); titers below LOD were all set to 5, all double-negative values were removed, and blue lines represent linear regression fit with 95% confidence interval (gray shading).

To investigate in-depth functional antibody responses to SARS-CoV-2 infection at convalescence, we employed two virus neutralization assays, one using an authentic live SARS-CoV-2 with a luciferase reporter (29) and another using a pseudovirus (PSV) neutralization assay (see Materials and Methods). We also measured total Ig binding to the spike protein RBD and NTD, as well as IgG binding to N protein antigen. We found that 98% (99/101) of donors generated antibodies to at least one SARS-CoV-2 antigen or virus (Fig. 1a and b), 92% (93/101) had at least two positive antibody assays, and 65% (65/101) had functional and binding antibodies to all viruses and antigens. Only two donors had negative results in every assay; both were asymptomatic and both were diagnosed by an antibody test. We found that the most sensitive assays to detect antibodies in recovered donors were the RBD total Ig assay (96% of donors positive), followed by PSV neutralization and N IgG assays (both 93% of donors positive).

All donors with undetectable RBD antibody titers also had undetectable neutralizing antibody assays, and the RBD total Ig and IgG binding assay showed the strongest correlation with the two neutralization assays (Fig. 1c to f and Fig. S2). Among the other binding assays, the N assay had the weakest correlations with both neutralization assays compared to the spike antigen-based NTD assay. We then looked at quantitative measures of functionally neutralizing as well as RBD-binding antibody levels by endpoint titer. The majority of donors (80%) had detectable live reporter virus-neutralizing antibody titers, and >50% of these exceeded 1:160 (the FDA-recommended threshold for therapeutic applications of convalescent-phase plasma) (Fig. S2). The majority of RBD total Ig and IgG endpoint titers were found to be within the range of 1:160 to 1:640, and overall the total Ig and IgG titers were very similar. Since isotype-specific IgA and, to a lesser extent, IgM antibodies may influence the early neutralizing antibody response (30), we also measured RBD IgA and IgM binding titers. Approximately 60% of donors demonstrated detectable IgA or IgM antibodies to RBD, with most in the lower titer range (1:20 to 1:159) (Fig. S2).

10.1128/mSphere.00275-21.2 FIG S2 Correlation plots and titers of antibody binding and functional assays. (a to d) Live reporter virus NT_50_ dilution plotted against antibody level. (e and f) Pseudovirus NT_50_ dilution plotted against antibody level. (g) NTD total Ig (index) plotted against nucleocapsid IgG (index value). (h and i) RBD total Ig antibody level (endpoint titer) plotted against antibody level. (j) Donor results by assay; titer ranges and percent shown. For panels a to i, Spearman’s rank correlation was used to calculate correlation coefficients (*r*) and *P* values (*p*); titers below LOD were set to 5, all double-negative values were removed, and blue lines represent linear regression with 95% confidence interval (gray shading). Download FIG S2, TIF file, 0.5 MB. Copyright © 2021 Markmann et al. 2021 Markmann et al. https://creativecommons.org/licenses/by/4.0/ This content is distributed under the terms of the Creative Commons Attribution 4.0 International license.

### Functional and binding antibody level durability.

**FIG 2:**
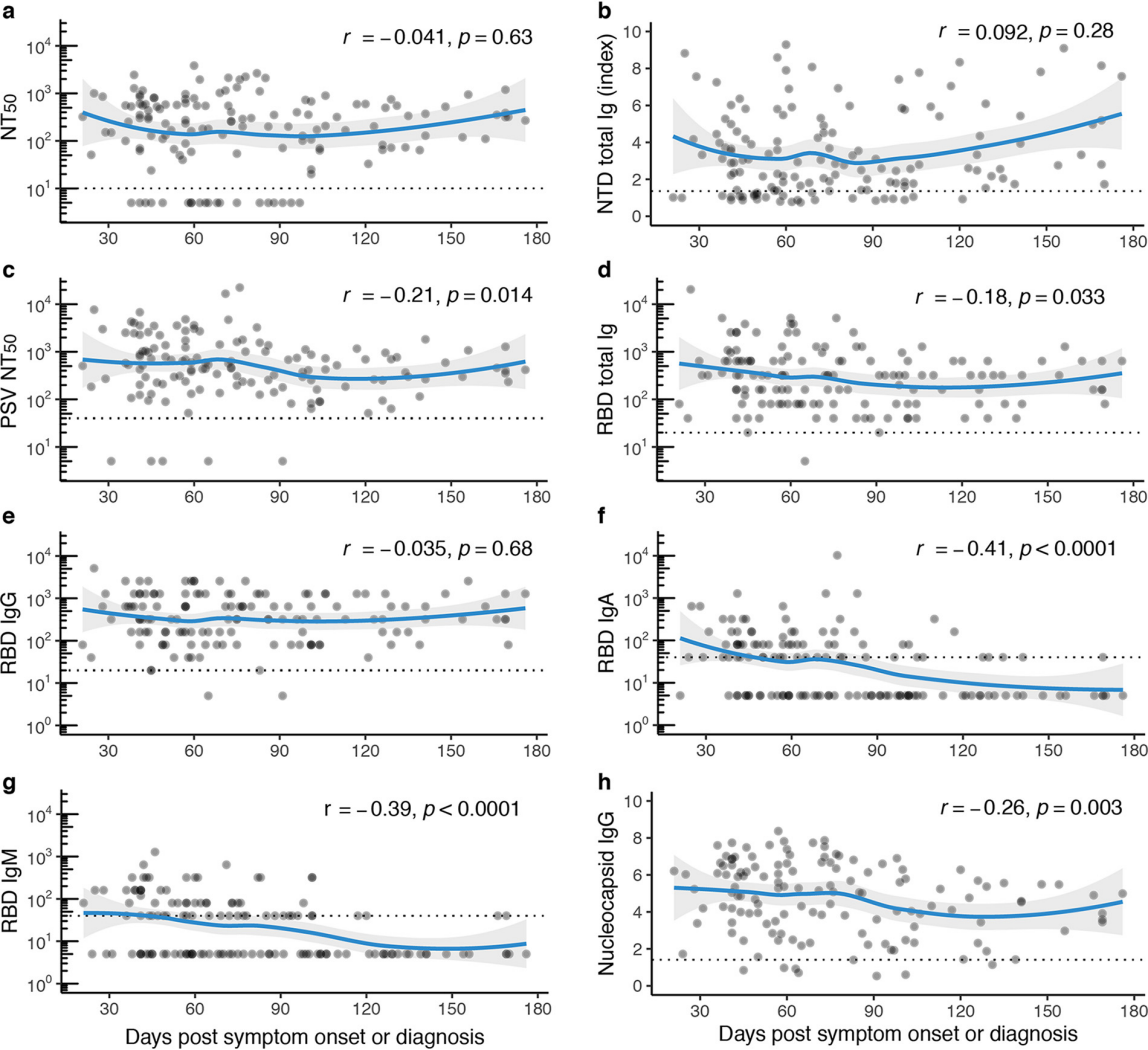
Antibody titers over time. (a) Functional antibody (NT_50_ dilution) plotted against days post-symptom onset or RT-PCR diagnosis; *r* = −0.041, *P* = 0.63. (b) NTD total Ig (index) plotted against days-post symptom onset or RT-PCR diagnosis; *r* = 0.092, *P* = 0.28. (c) Functional antibody (PSV NT_50_ dilution) plotted against days post-symptom onset or RT-PCR diagnosis; *r* = −0.21, *P* = 0.014. (d) RBD total Ig (endpoint titer) plotted against days post-symptom onset or RT-PCR diagnosis; *r* = −0.18, *P* = 0.033. (e) RBD IgG (endpoint titer) plotted against days post-symptom onset or RT-PCR diagnosis; *r* = −0.035, *P* = 0.68. (f) RBD IgA (endpoint titer) plotted against days post-symptom onset or RT-PCR diagnosis; *r* = −0.41, *P* < 0.0001. (g) RBD IgM (endpoint titer) plotted against days post-symptom onset or RT-PCR diagnosis; *r* = −0.39, *P* < 0.0001. (h) Nucleocapsid IgG (index) plotted against days post-symptom onset or RT-PCR diagnosis; *r* = 0.092, *P* = 0.0029. For panels a to h, *n* = 138, nonparametric, two-tailed Spearman’s rank correlation was used to calculate correlation coefficients (*r*) and *P* values (*p*); titers below LOD were set to 5, all double-negative values were removed, and blue lines represent LOESS regression fit with 95% confidence interval (gray shading).

Overall donor antibody levels, including additional donations from 31/101 donors who donated more than once (total samples donated *n* = 149), revealed stable neutralizing, RBD, and NTD-binding antibodies over 6 months (Fig. 2). Among the specific assays, neutralizing antibodies to virus and binding antibodies to NTD were the most stable out to 180 days (Fig. 2a and b). Through 120 days and beyond, there was a slight decrease in PSV-neutralizing antibodies and total Ig binding antibodies to RBD (Fig. 2c and d). RBD total Ig decreases over time were likely due in part to the decline of IgA and IgM titers that we observed after day 90 (Fig. 2f and g) but not due to shifts in RBD IgG levels (Fig. 2e). Notably, compared with antibodies directed against spike protein antigens, there was a stronger decrease in N binding IgG levels over this time period (Fig. 2h). When comparing the correlation coefficients of the trendlines in Fig. 2a and d to h with a Fisher r-to-z transformation, we found significant (*P* < 0.05) differences only between NTD antibody levels compared to all of the other assays which either stayed constant or decreased.

We then studied in detail the donors who provided sequential donations to examine temporal kinetics of antibody levels at an individual level. Overall functional neutralizing antibody levels to live reporter virus and RBD-binding Ig levels showed no significant changes between donation times (Fig. S3). To ascertain if initial antibody titer plays a role in antibody changes over time, we separated sequential donors into three groups by initial titer: >1:640, 1:160 to 1:640, and 1:20 to 1:159. Median live reporter viral neutralization antibody titers (Fig. S3i to k) and RDB Ig antibody titers (Fig. S3l to n) between the first two donations showed a modest decrease in the highest-initial-titer group (>1:640) but not in the lower-titer groups. However, earlier time points are needed in the lower-titer groups to better compare these levels to the high-titer group, as we may not see changes in the lower-titer groups due to longer time to first donation in these groups. This decrease in the RBD total Ig group with initial titer >1:640 was likely due to RBD IgA and IgM levels in these donors, which showed a significant decline between the first two donations (*P* < 0.05) (data not shown).

10.1128/mSphere.00275-21.3 FIG S3 Antibody levels in sequential donors. (a) Functional antibody (NT_50_ dilution) of sequential donors over four donations. (b) NTD total Ig (index) of sequential donors over four donations. (c) Pseudovirus NT_50_ dilution of sequential donors over four donations. (d) RBD total Ig titers of sequential donors over four donations. (e) RBD IgG (endpoint titer) of sequential donors over four donations. (f) RBD IgM (endpoint titer) of sequential donors over four donations. (g) RBD IgA (endpoint titer) of sequential donors over four donations. (h) Nucleocapsid IgG (index value) of sequential donors over four donations. (i to k) Functional antibody (NT_50_ dilution) stratified by titer levels at first donation. (l to n) RBD total Ig stratified by titer levels at first donation. Titers are presented as geometric mean with geometric coefficient of variation. Statistical significance was determined using nonparametric Kruskal-Wallis test adjusted for multiple comparisons for panels a to h. For panels i to n, statistical significance was determined using Mann-Whitney U tests comparing donation 1 with donation 2 for which matching donor data were available. Download FIG S3, TIF file, 0.6 MB. Copyright © 2021 Markmann et al. 2021 Markmann et al. https://creativecommons.org/licenses/by/4.0/ This content is distributed under the terms of the Creative Commons Attribution 4.0 International license.

### Demographic and clinical correlates of functional antibody titers.

**FIG 3:**
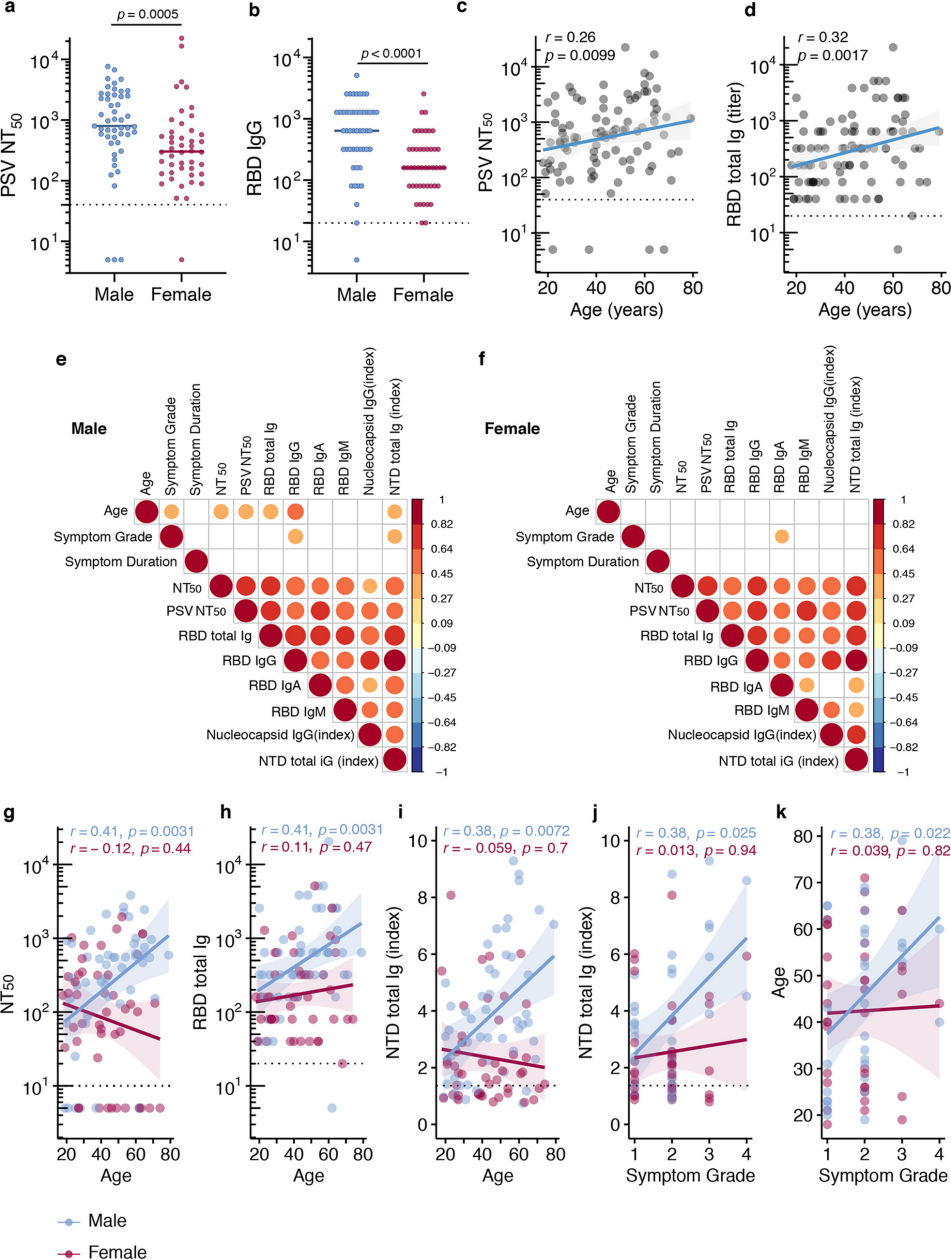
Clinical correlates of antibody titers. (a) Functional antibody (PSV NT_50_ dilution) in males (*n* = 49) and females (*n* = 46) at first donation. (b) RBD IgG titers in males and females at first donation. Horizontal bars indicate median values. For panels a and b, statistical significance was determined using Mann-Whitney U tests. (c and d) Spearman’s correlation between age and PSV NT_50_ or RBD Ig levels at first donation. (e and f) Nonparametric, two-tailed, Spearman’s correlation heat map of clinical correlates and antibody titers stratified by sex (red = positive association, blue = negative association, blank = nonsignificant association). (g to k) Correlation between age and NT_50_ or RBD Ig levels at first donation. Spearman’s rank correlation was used to calculate correlation coefficients (*r*) and *P* values (*p*).

SARS-CoV-2 binding and functionally neutralizing antibody levels were higher in males than in females (Fig. 3a and b and Fig. S4), increased with increasing age, and correlated positively with male age and symptom grade (Fig. 3c and d). This difference between males and females (Fig. 3a and b) remained after negative data points were removed from each analysis. Surprisingly, positive correlations with antibody levels and age and symptom grade (Fig. 3e to k and Fig. S5a to f) were restricted to the male population (Fig. 3e and f). Sex stratification revealed that in males, age and symptom grade were significantly positively correlated, as were age and RBD Ig and functionally neutralizing antibody levels (Fig. 3e and g to k). On the other hand, in females, only RBD IgA levels were associated with symptom grade (Fig. 3f). Males and females were equally likely to be hospitalized (*P* = 0.95).

10.1128/mSphere.00275-21.4 FIG S4 Additional demographic correlates of neutralization. (a to f) Binding and neutralizing antibodies in males and females at first donation. (g to l) Spearman correlation between age and binding and neutralizing antibodies at first donation. (m to t) Binding and neutralizing antibodies in donors with no reported comorbidities, one comorbid condition, and two or more comorbidities. For panels a to f and m to t, horizontal bars indicate median values. Statistical significance was determined using Mann-Whitney U tests. For panels g to l, Spearman’s rank correlation was used to calculate correlation coefficients (*r*) and *P* values (*p*), and blue lines represent linear regression fit with 95% confidence interval (gray shading). Download FIG S4, TIF file, 0.5 MB. Copyright © 2021 Markmann et al. 2021 Markmann et al. https://creativecommons.org/licenses/by/4.0/ This content is distributed under the terms of the Creative Commons Attribution 4.0 International license.

10.1128/mSphere.00275-21.5 FIG S5 Symptom correlates of binding and neutralizing antibodies. (a to h) Spearman’s correlation between symptom grade and neutralizing or binding antibody level at first donation. (i to p) Spearman’s correlation between symptom duration and neutralizing or binding antibody level at first donation. Spearman’s rank correlation was used to calculate correlation coefficients (*r*) and *P* values (*p*). Download FIG S5, TIF file, 0.5 MB. Copyright © 2021 Markmann et al. 2021 Markmann et al. https://creativecommons.org/licenses/by/4.0/ This content is distributed under the terms of the Creative Commons Attribution 4.0 International license.

**FIG 4:**
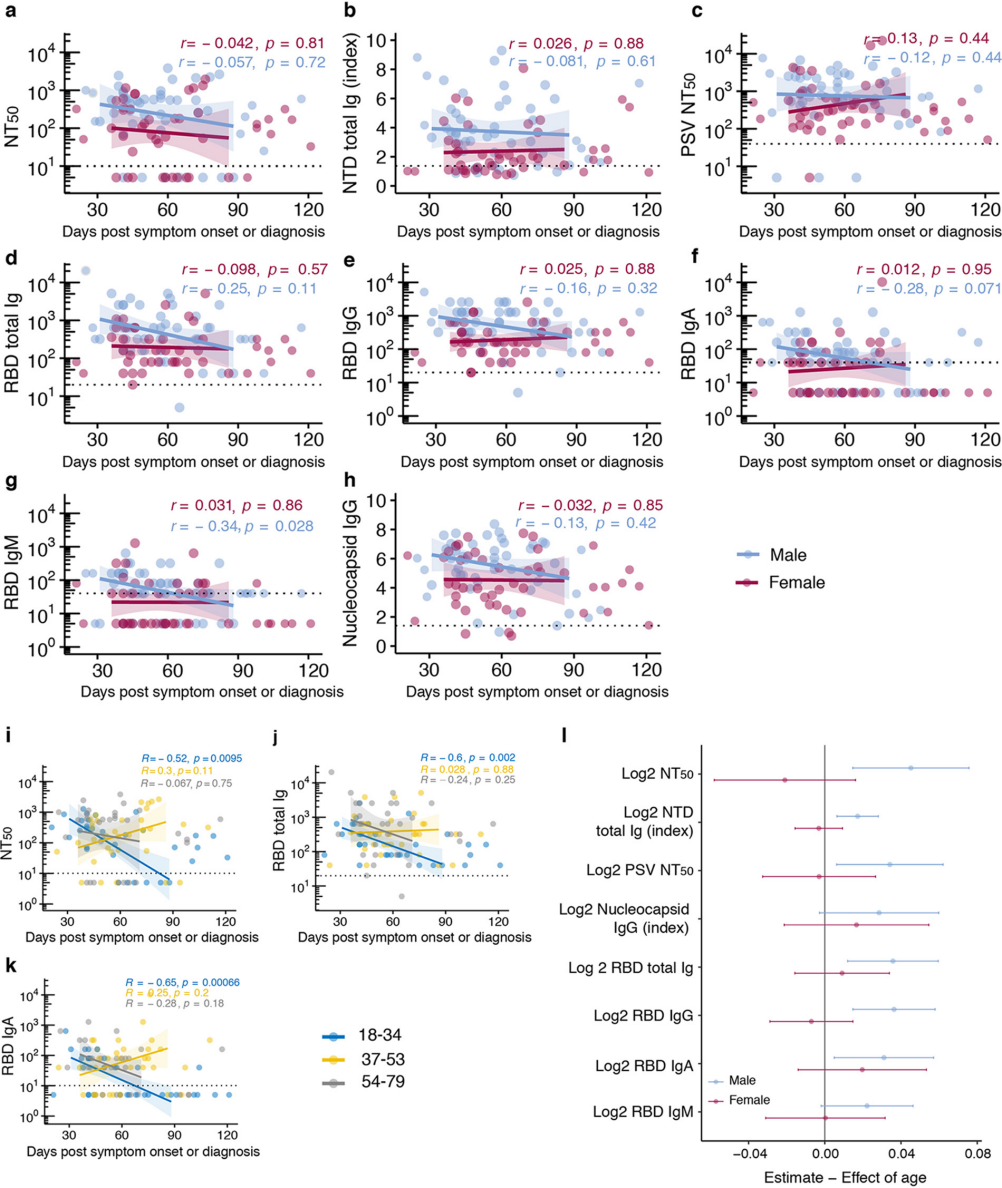
Antibody differences between sexes and age groups. (a to h) Differences in (a) functional antibody (NT_50_ dilution) levels, (b) NTD total Ig (index), (c) functional antibody (PSV NT_50_ dilution) levels, (d) RBD total Ig (endpoint titers), (e) RBD IgG (endpoint titers), (f) RBD IgA (endpoint titers), (g) RBD IgM, and (h) nucleocapsid IgG (index) between males (*n* = 49) and females (*n* = 46) at first donation. (i) Differences in functional antibody (NT_50_ dilution) levels between age groups. (j) Differences in RBD total Ig titers between age groups. (k) Differences in RBD IgA (endpoint titers) between age groups. For panels i to k, donors were divided into tertiles based on their age. For panels a to h, lines represent linear regression fit and shaded areas represent 95% confidence interval. Lines from linear regression were fitted from days 30 to 90 to avoid overfitting where fewer observations were available. Spearman’s rank correlation was used to calculate correlation coefficients (*r*) and *P* values (*p*). (l) Forest plot of estimated effect (95% confidence interval) of age on antibody titers at first donation, stratified by sex. Linear regression model was adjusted for time from symptom onset or RT-PCR diagnosis.

We then examined the possibility that antibody stability over time was influenced by sex or age. No significant differences were observed in neutralizing or binding antibody levels over time (first 90 days) between males and females (Fig. 4a to h). In contrast, there were rapid declines in both types of antibodies in the youngest age group (18 to 43 y/o) over the first 90-day period (Fig. 4i and j) that may have been related to decreases in serum RBD IgA, but not IgM, which showed a significant decline in this age group over this time period (Fig. 4k). We then calculated an estimate of the effect of age, adjusted for time from symptom onset to donation, stratified by sex on the various functional and binding antibody levels. Among males, we observed that for each 1-year increase in age there was a significant increase in antibody levels in all assays tested except N IgG and RBD IgM, but among females, age did not seem to affect antibody levels after accounting for time from symptom onset (Fig. 4l).

Since we identified that in male donors, increased symptom grade or disease severity correlated with higher antibody levels, we looked more closely at individual symptoms to ascertain if any in particular were associated with each other or with donor serum antibody levels. We found that of the most common symptoms, only loss of sense of taste and smell were associated, though more strongly in female than male donors (Fig. S6a and b). Surprisingly, we found a negative effect of reporting tiredness or fatigue in male donors on the level of RBD Ig binding antibodies (Fig. S6b). We also evaluated the association between antibody levels and the presence of comorbid conditions and found that donors with cardiometabolic diseases had higher levels of neutralizing, RBD Ig, N IgG, and NTD Ig antibodies (Fig. S7). This observation was independent of sex. Symptom duration (Fig. 3 and Fig. S5h to n), nulliparity, and ABO blood group were not significantly associated with functional or binding antibody levels.

10.1128/mSphere.00275-21.6 FIG S6 Symptomatology between sexes. (a) Heat map of phi coefficients examining the interrelationship among reported symptoms in males (*n* = 46) and females (*n* = 44). Green indicates positive association, and pink indicates negative association. (b) Forest plot showing the effect of reported symptoms on the levels of live reporter virus-neutralizing antibodies (left panel) and RBD total Ig (right panel) in males and females. Effects were calculated using linear regression models adjusted for age and time from symptom onset or PCR diagnosis. Download FIG S6, TIF file, 0.2 MB. Copyright © 2021 Markmann et al. 2021 Markmann et al. https://creativecommons.org/licenses/by/4.0/ This content is distributed under the terms of the Creative Commons Attribution 4.0 International license.

10.1128/mSphere.00275-21.7 FIG S7 Antibody levels in donors with reported comorbid conditions. Donors were categorized into individuals with or without hay fever and/or asthma and into individuals with or without cardiometabolic conditions (diabetes, obesity, hypertension, cardiovascular disease). Horizontal bars indicate median values. Statistical significance was determined within the two groups of conditions using Mann-Whitney U tests. Download FIG S7, PDF file, 0.3 MB. Copyright © 2021 Markmann et al. 2021 Markmann et al. https://creativecommons.org/licenses/by/4.0/ This content is distributed under the terms of the Creative Commons Attribution 4.0 International license.

## DISCUSSION

In-depth serological, clinical, and demographic correlates of durable and protective functional antibodies in individuals who have recovered from COVID-19 have not been well described. Understanding serological responses to COVID-19 disease and vaccination will allow us to define which antibody populations may be protective against reinfection and thus act as immunological correlates of protection. Of 101 convalescent-phase plasma donors who experienced a range of COVID-19 disease, the vast majority have detectable levels of functionally neutralizing as well as binding antibodies to SARS-CoV-2 RBD, NTD, and N antigens. Furthermore, though their titers are heterogeneous, most donors have neutralizing and RBD-targeting antibody titers of >1:160. Even low levels of functionally neutralizing antibodies to SARS-CoV-2, as seen here in about a quarter of donors, are protective in nonhuman primate vaccine models (31, 32). This suggests that low serum levels of a few highly potent antibodies may be enough to confer protection, and we find that such antibodies are nearly universally produced upon exposure to the virus in this donor cohort of mostly symptomatic cases.

Of three SARS-CoV-2 antigens used for antibody detection in this study, the RBD was the most sensitive in detecting prior SARS-CoV-2 infection. Furthermore, RBD total Ig levels showed the strongest correlation with functionally neutralizing antibodies, suggesting its role as the immunodominant antigenic target of antibodies that neutralize SARS-CoV-2 infection. We found that 95% of sera with an RBD total Ig titer of ≥1:160 had positive live reporter virus-neutralizing antibody titers, suggesting that this may be a cutoff used as a surrogate for functional antibody assays. Furthermore, the majority of donors with undetectable live reporter virus-neutralizing antibody levels had detectable RBD-binding antibodies, suggesting they may have RBD-targeting neutralizing antibodies that are below the assay detection limit. This hypothesis can be tested in future studies using passive transfer mouse protection models. This highlights the potential role for RBD-based antibody assay development and testing as a surrogate for functional antibody assays that could be deployed in the clinical and vaccination setting in a scalable, high-throughput fashion.

The strongest demographic correlate of neutralizing antibody levels we found was male sex. Studies have shown that COVID-19 disease is associated with higher morbidity and mortality rates in men than in women (33). The reason for this finding is unknown and seems unrelated to CD8^+^ and CD4^+^ T cell frequency (22); however, it may be related to an uncoordinated response between CD4^+^ T cell responses and serum antibody responses driven by the presence of underlying comorbidities (34). Another recent finding suggests higher thromboembolism risk in males than in females (35). Sex differences in other respiratory viral disease outbreaks have been seen, for example, during the 2009 influenza pandemic where female sex correlated with severe disease in a young cohort in Canada (36). Some viral infections as well as vaccinations such as the influenza vaccination have been seen to elicit stronger serum antibody and cellular immune responses in females (37), while others elicit stronger serologic antibody responses in males (38). Differences in disease severity and humoral responses to vaccines have been hypothesized to be influenced by a combination of sex hormone effects on immune cell signaling, X chromosome immune-related gene expression and microRNA (miRNA) levels, and genetic polymorphisms (37) in genes encoding important immunologic proteins such as interleukin-6 (IL-6) (39) and cytotoxic-T-lymphocyte-associated antigen 4 (CTLA-4) (40).

Significantly higher SARS-CoV-2 RBD antibodies and functionally neutralizing antibodies in male than in female COVID-19 CP donors with mild to moderate disease in the first 30 to 114 days post-symptom onset have been previously reported (23–28). Our findings support these data and add that these sex differences in antibody levels are also seen with SARS-CoV-2 N protein and NTD antigens and that age and symptom grade also influenced the sex disparity in RBD-binding and functionally neutralizing antibody responses. We further add that independent of sex, donors with cardiometabolic diseases had higher levels of neutralizing, RBD Ig, N IgG, and NTD Ig antibodies than those without cardiometabolic diseases. These findings confirm male sex, especially males with increased age and worse COVID-19 symptom severity, as a demographic correlate of functional antibodies and symptom severity as well as cardiometabolic disease as clinical correlates of functional antibodies. Our findings, however, contrast with other mild disease cohort studies out to 60 to 125 days post-symptom onset where binding and/or neutralizing antibody levels were higher in females (41–43) or showed no difference between males and females (44–49); these data were recently reviewed elsewhere (50). These differences may be due to differences in sampling methods, cohort comorbidities which are not usually identified, and/or geography. Our findings recognize that there are currently unknown underlying factors which predispose older males and individuals with cardiometabolic disease to either prolonged viral replication and immune exposure to SARS-CoV-2 or differential immune activation.

We do not yet know what level of functional antibodies is required for protection from SARS-CoV-2. Although female donors in this cohort have lower antibody levels than males, this may be enough to confer long-term protection. This observation warrants further investigation, including consideration of a similar sex bias in vaccine-induced immunity. Furthermore, we found significant differences in sex and functional antibody production despite reported disease severity, suggesting that prolonged viremia and/or abnormal cytokine activation may not be the only things responsible for this finding. Other hypotheses that have been made to explain COVID-19 disease sex differences include poorer T cell responses in males (33) and the presence of previously undetected autoantibodies against t I interferons (51) in males with severe disease. On the other hand, the hypothesis that expression of ACE2 and TMPRSS, important SARS-CoV-2 cellular entry receptors in human lung and other tissues, plays a role in the sex disparity is thought to be an unlikely explanation (52).

In the face of SARS-CoV-2 vaccinations and new viral mutations, it is critical to define functional antibody durability after natural infection and vaccination. Here, we show that functionally neutralizing antibodies to live SARS-CoV-2 virus remain stable months post-symptom onset and that this is likely maintained to 180 days. Not surprisingly, levels of RBD-binding IgA and IgM antibodies declined rapidly within the first 3 months after symptom onset. However, NTD-binding Ig antibodies remain stable, and RBD-binding Ig antibodies declined modestly. Levels of neutralizing and RBD Ig antibodies on an individual level were also maintained, with only a modest decrease within the first 90 days after symptom onset in donors with initial titers of >1:640. When broken down by age group, 18- to 34-year-old donors demonstrated a significant decrease in functional antibody and RBD Ig responses over the first 90 days post-symptom onset that was likely driven by rapidly declining RBD IgA levels.

We also find that N IgG antibodies correlate least with neutralizing antibodies and continue to decline 120 to 180 days post-symptom onset, a trend which was noted 90 days post-symptom onset in a mild-disease community cohort (18). This suggests that though SARS-CoV-2 N antibodies may be generated at high levels early after symptomatic infection, N may not be an immunodominant target of the adaptive immune response and thus is a less sensitive measure of remote infection. This further suggests that the use of N protein in seroprevalence studies may bias results toward more recent infections and warrants further investigation in cohorts of mild and asymptomatic COVID-19 disease.

One major limitation of this study is the demographic uniformity of our study population, which limits the generalizability of our findings and highlights the need to do these studies with a more diverse and representative population. Another bias in our donor population is our focus on recalling donors with higher neutralizing antibody titers to repeat donations. Thus, our “sequential donation” population is biased toward higher-titer donors.

Understanding human antibody responses and correlates of neutralizing antibodies to SARS-CoV-2 is critical in the next coming phase of understanding SARS-CoV-2 vaccine efficacy and protection against reinfection. We find that SARS-CoV-2 functionally neutralizing antibodies are maintained for months after infection. Our findings further support the role of RBD-binding antibodies as correlates of functionally neutralizing antibodies, suggesting that vaccines that induce potent RBD responses may be particularly efficacious. Furthermore, we add to the growing literature a role for sex as a correlate of SARS-CoV-2 functional neutralization. The association of male sex in this cohort with higher neutralizing antibody levels reveals a sexual dimorphism in humoral immune responses to SARS-CoV-2. We hypothesize that this is likely due to a combination of factors such as differences in duration of mucosal replication, T cell responses, sex hormone roles in immune activation, and genetic differences in immune responses. This finding may have clinical as well as vaccine outcome implications and warrants further investigation.

## MATERIALS AND METHODS

### Donors and plasma collection.

Convalescent-phase plasma was obtained from volunteer donors who met U.S. Food and Drug Administration (FDA) criteria for plasma collections in the UNC Blood Donor Center. Donors were recruited via Internal Review Board (IRB)-approved direct contact of SARS-CoV-2-positive persons diagnosed through the hospital laboratory system and public solicitation through multiple media outlets. Fresh sera and plasma collected in the diversion pouch as part of the standard plasmapheresis procedure were saved for research from donors who consented to study participation. All donors had confirmed SARS-CoV-2 infection by blood antibody testing or nasopharyngeal swab indicating the presence of SARS-CoV-2 RNA as performed by reverse transcriptase PCR (RT-PCR) in a U.S. laboratory with a Clinical Laboratory Improvement Amendments certification. All donors were recovered from their COVID-19 illness and qualified for collection in adherence with FDA regulatory guidance. As required at the time, some donors had a negative repeat SARS-CoV-2 RT-PCR test done within 72 h prior to donation. At the time of plasma collection, donors were offered participation in the study. All donors who participated provided written informed consent. The research was approved by the UNC Institutional Review Board and conducted under good clinical research practices. Participating donor characteristics and information regarding COVID-19 symptoms and history were obtained through in-person and telephone interviews using a standardized questionnaire as part of UNC IRB no. 20-1141. We used RStudio (R version 3.6.2) (53) to generate a map of the counties our donors reside in. We generated a 4-point symptom severity scale for this study based on the National Institutes of Health Division of AIDS grading system (54). For this study time period, we did not prescreen donors to determine the presence of SARS-CoV-2 antibodies; donor qualifications were based strictly on their positive SARS-CoV-2 diagnostic test and eligibility for plasma donation.

### Recombinant SARS-CoV-2 spike protein antigens.

The production of RBD antigen from SARS-CoV-2 was previously described (3). The NTD antigen (16 to 305 amino acids, accession no. P0DTC2.1) was cloned into the pαH mammalian expression vector and purified using nickel-nitrilotriacetic acid agarose in the same manner.

### ELISAs.

The RBD enzyme-linked immunosorbent assay (ELISA) used in this study was initially described previously (3), and the NTD ELISA was performed in the same manner. Briefly, ELISAs were done either as a single-point dilution at 1:40 or as serial titrations starting at a dilution of 1:20 or 1:40. ELISA plates were coated with 200 ng/well of antigen and blocked, a 2-fold serum dilution series was done, and diluted serum was incubated for 1 h at 37°C. Alkaline phosphatase-linked secondary antibodies were used at 1:2,500 dilution each (IgM and IgG, Sigma; IgA, Abcam). The PNPP substrate (Sigma) was added to develop the plate, and absorbance was measured at 10 min for total Ig (combination of all three secondary antibodies) or IgG or 25 min for IgA or IgM at 405 nm using a plate reader (BioTek). Each sample was tested in duplicate. Antibody titration measurements were recorded as endpoint titers. Ten plasma samples were tested in the RBD total Ig format and compared to serum; all titer results were within a 2-fold dilution (data not shown). Receiver operating characteristic analyses were done to obtain cutoff values and sensitivity and specificity estimates on the SARS-CoV-2 assays using pre-2019 negative-control sera and RT-PCR-confirmed COVID-19 cases that were at least 9 days post-symptom onset (see Table S3 in the supplemental material). Positive and negative controls were used to standardize each ELISA and normalize across experiments.

10.1128/mSphere.00275-21.10 TABLE S3 Binding assay receiver operating characteristic (ROC) analysis results. Download Table S3, DOCX file, 0.1 MB. Copyright © 2021 Markmann et al. 2021 Markmann et al. https://creativecommons.org/licenses/by/4.0/ This content is distributed under the terms of the Creative Commons Attribution 4.0 International license.

### Nucleocapsid protein ELISA.

Detection of IgG antibody to SARS-CoV-2 N antigen was performed with a microparticle chemiluminescence assay (Abbott Laboratories) on the Abbott Architect i2000SR immunoassay analyzer. The EUA-approved Abbott SARS-CoV-2 IgG assay utilizes microparticles coated with SARS-CoV-2 N protein to capture N-specific IgG. Bound IgG was detected via addition of anti-human acridinium-labeled second-step antibody. Following a second wash step, pretrigger and trigger solutions were added, and a chemiluminescent reaction was detected and reported in relative light units (RLU). The RLU generated are reflective of the amount of antibody bound to the microparticles. The sample RLU were compared to the assay-specific calibrator RLU to generate an index value (S/C). Index values of ≥ 1.4 were considered positive. Sensitivity and specificity have been previously obtained for this assay (Table S3) (55, 56).

### SARS-CoV-2-WA1 neutralization assay.

Full-length SARS-CoV-2 viruses expressing a nanoluciferase gene were designed and recovered via reverse genetics as previously described (3, 29) in a 96-well microneutralization format. Briefly, Vero E6 cells were infected with SARS-2-nLuc viruses, and titers were determined to generate an 8-point curve. Initial serum dilutions to detect the presence of neutralizing antibody were 1:20 or 1:50, and all serum samples were tested in duplicate. Internal serum controls, cell-only controls, and virus-only controls were included in each neutralization assay plate. Plates were incubated for 48 h, at which point cells were lysed and luciferase activity was measured on a Nano-Glo luciferase assay system (Promega). Antibody neutralization titers to SARS-CoV-2 were reported as serum dilutions at which a 50% reduction in relative light units (NT_50_) to virus-only controls was observed. Limit of detection (LOD) was set to 1:10, or one-half the starting dilution of 1:20, since all NT_50_ values above a titer of 1:10 that were run with a 1:50 starting dilution were >1:25. Thirteen plasma samples were tested and compared to serum; all NT_50_ results were within a 3-fold dilution (data not shown). Pre-COVID-19 serum samples were also tested, and 13/13 had an NT_50_ of <1:20 in this assay.

### SARS-CoV-2 pseudovirus neutralization assay.

The “PhenoSense SARS CoV-2-neutralizing antibody (NAb) assay” has been developed by leveraging the proprietary PhenoSense assay platform that was developed to evaluate antiretroviral drug susceptibility (57) and later adapted to evaluate entry inhibitors and neutralizing antibody (58) as well as coreceptor tropism (59). The production of luciferase is dependent on virus entry and the completion of a single round of virus replication. Agents that inhibit pseudovirus entry or replication reduce luciferase activity in a dose-dependent manner, providing a quantitative measure of drug and antibody susceptibility.

The measurement of neutralizing antibody activity using the PhenoSense SARS CoV-2 NAb assay is performed by generating HIV-1 pseudovirions that contain and express the complete SARS-CoV-2 spike protein open reading frame. The pseudovirus is prepared by cotransfecting HEK293 producer cells with an HIV-1 genomic vector and a SARS-CoV-2 envelope expression vector. Neutralizing antibody activity is measured by assessing the inhibition of luciferase activity in HEK293 target cells expressing the ACE2 receptor following preincubation of the pseudovirions with serial dilutions of the serum specimen. The expression of luciferase activity in target cells is inhibited in the presence of anti-SARS-CoV-2 neutralizing antibody. Data are displayed by plotting the percent inhibition of luciferase activity against log_10_ reciprocal of the serum/plasma dilution. Neutralizing antibody titers are reported as the reciprocal of the serum dilution conferring 50% inhibition (NT_50_) of pseudovirus infection.

$$\text{\%}\;\text{Inhibition }=\;100\text{\% }\;–\;\left(\left(\frac{\text{RLU}\left(\text{Vector}\;+\;\text{Sample}\;+\;\text{Diluent}\right)\;\text{– RLU}\left(\text{Background}\right)}{\text{RLU}\left(\text{Vector}\;+\;\text{Diluent}\right)\;\text{– RLU}\left(\text{Background}\right)}\right)\;\times \;100\%\right)$$

The results of the PhenoSense SARS CoV-2 NAb assay can be reported as an NT_50_ titer (1/dilution) or qualitatively (positive, negative) based on a predefined dilution cutoff (e.g., >50% inhibition at 1:40 dilution). To ensure that the measured neutralizing antibody activity is SARS-CoV-2 specific, each test specimen is also assessed using a nonspecific pseudovirus (specificity control) that expresses a nonreactive envelope protein of one or more unrelated viruses (e.g., avian influenza virus).

### Statistical analyses.

We used the Wilcoxon rank sum test to test for differences between two groups and the Kruskal-Wallis test followed by Benjamini-Yekutieli correction to test for differences between three or more groups. We calculated the phi coefficient as a measure of association between two binary factors and relied on the chi-square test to test for differences. We also calculated the Spearman rank correlation coefficient and used locally estimated scatterplot smoothing (LOESS) to visualize antibody trends over time. Linear regression models were used to further assess relationships with antibody levels, after first transforming antibody levels to the base-2 logarithm scale. Venn diagram and correlation heat maps were created to visualize associations. All statistical analyses were performed using R 4.0.2 (Vienna, Austria), all tests were two sided, and a *P* value of <0.05 was considered statistically significant.
