# Erratum for Markmann et al., “Sex Disparities and Neutralizing-Antibody Durability to SARS-CoV-2 Infection in Convalescent Individuals”

**DOI:** 10.1128/mSphere.00736-21

**Published:** 2021-09-08

**Authors:** Alena J. Markmann, Natasa Giallourou, D. Ryan Bhowmik, Yixuan J. Hou, Aaron Lerner, David R. Martinez, Lakshmanane Premkumar, Heather Root, David van Duin, Sonia Napravnik, Stephen D. Graham, Quique Guerra, Rajendra Raut, Christos J. Petropoulos, Terri Wrin, Caleb Cornaby, John Schmitz, JoAnn Kuruc, Susan Weiss, Yara Park, Ralph Baric, Aravinda M. de Silva, David M. Margolis, Luther A. Bartelt

**Affiliations:** a Department of Medicine, Division of Infectious Diseases, University of North Carolina School of Medicine, Chapel Hill, North Carolina, USA; b Centre of Excellence in Biobanking and Biomedical Research, Molecular Medicine Research Center, University of Cyprus, Nicosia, Cyprus; c Department of Microbiology and Immunology, University of North Carolina School of Medicine, Chapel Hill, North Carolina, USA; d Department of Epidemiology, University of North Carolina at Chapel Hill, Chapel Hill, North Carolina, USA; e Department of Medicine, University of North Carolina School of Medicine, Chapel Hill, North Carolina, USA; f LabCorp-Monogram Biosciences, South San Francisco, California, USA; g Department of Pathology & Laboratory Medicine, University of North Carolina School of Medicine, Chapel Hill, North Carolina, USA; h UNC HIV Cure Center, University of North Carolina School of Medicine, Chapel Hill, North Carolina, USA

## ERRATUM

Volume 6, no. 4, e00275-21, 2021, https://doi.org/10.1128/mSphere.00275-21. Fig. 2: in the legend for panel h, the *r* value should be −0.26, not 0.092.

Fig. 3: the first sentence of the legend for panels g to k should read “(g to k) Correlation between age or symptom grade and NT_50_ or RBD Ig levels at first donation.”

Acknowledgments: in the third paragraph, the author initials X.J.H. and P.L. should be Y.J.H. (Yixuan J. Hou) and L.P. (Lakshmanane Premkumar), respectively.

