# Supplementary figures and images for: Sex Disparities and Neutralizing-Antibody Durability to SARS-CoV-2 Infection in Convalescent Individuals
Source: mSphere. 2021 Aug 25;6(4):e00275-21. doi: 10.1128/mSphere.00275-21 (PMC8386415; doi:10.1128/mSphere.00275-21)

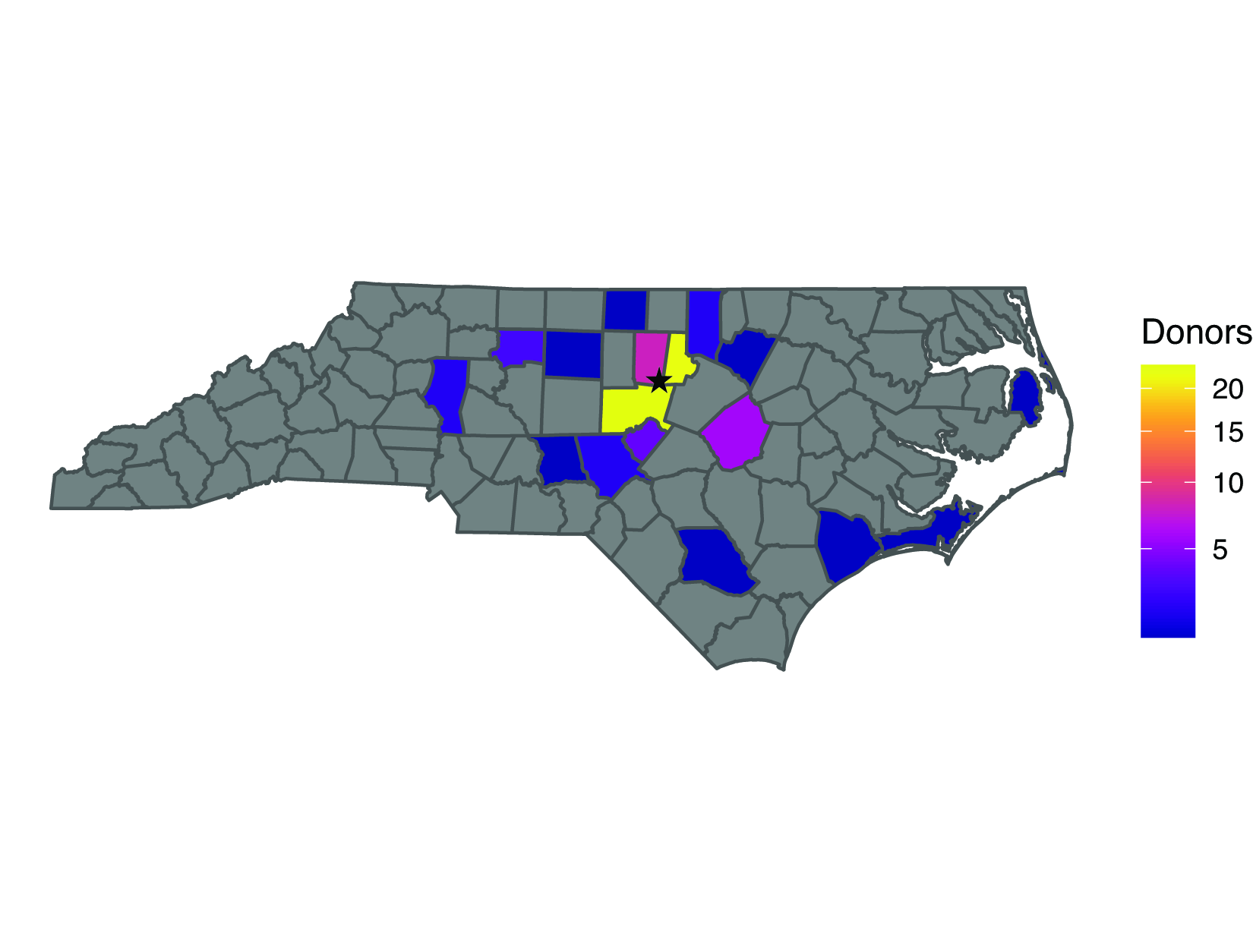

Supplement: FIG S1 [file msphere.00275-21-sf001.tif]

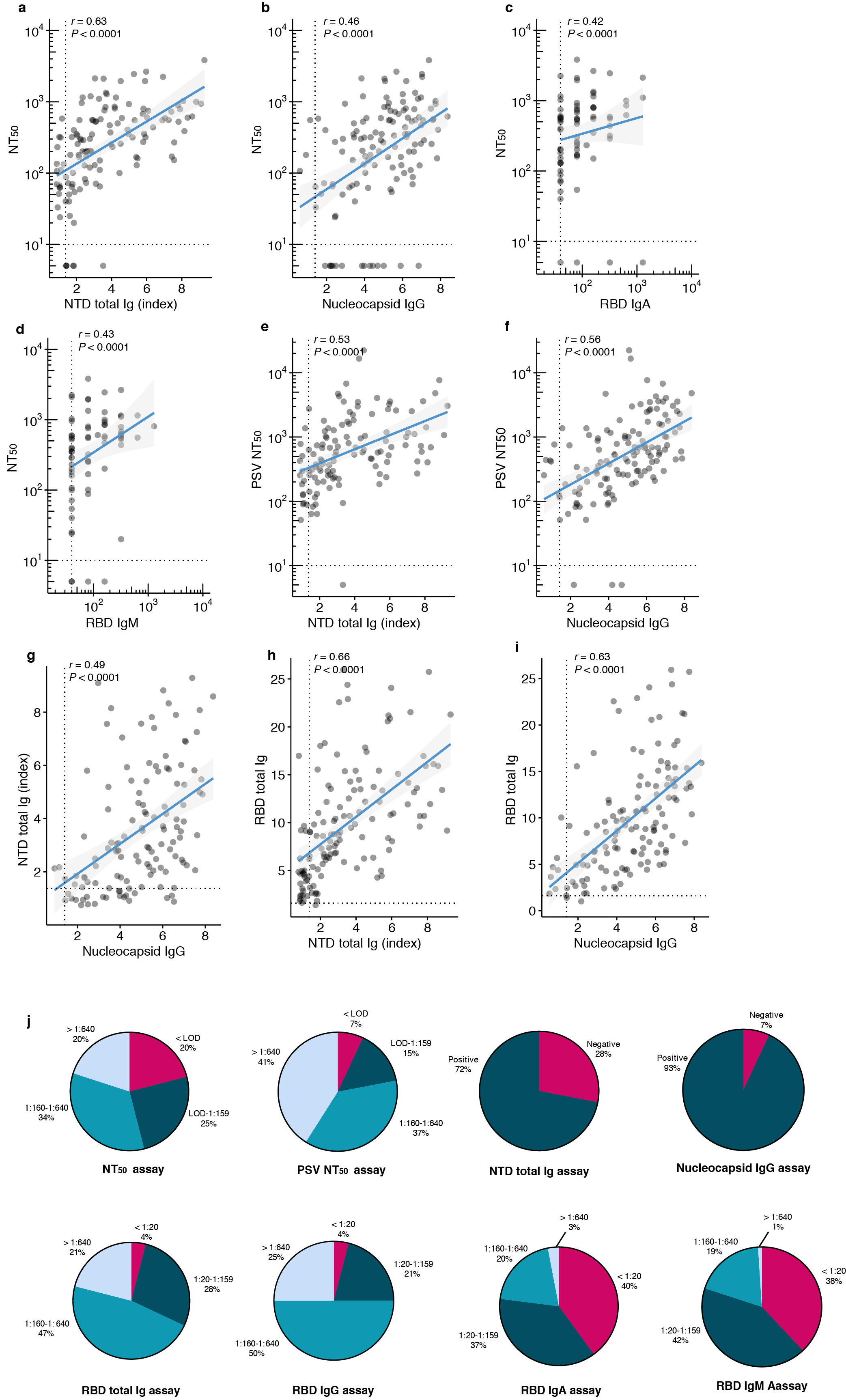

Supplement: FIG S2 [file msphere.00275-21-sf002.tif]

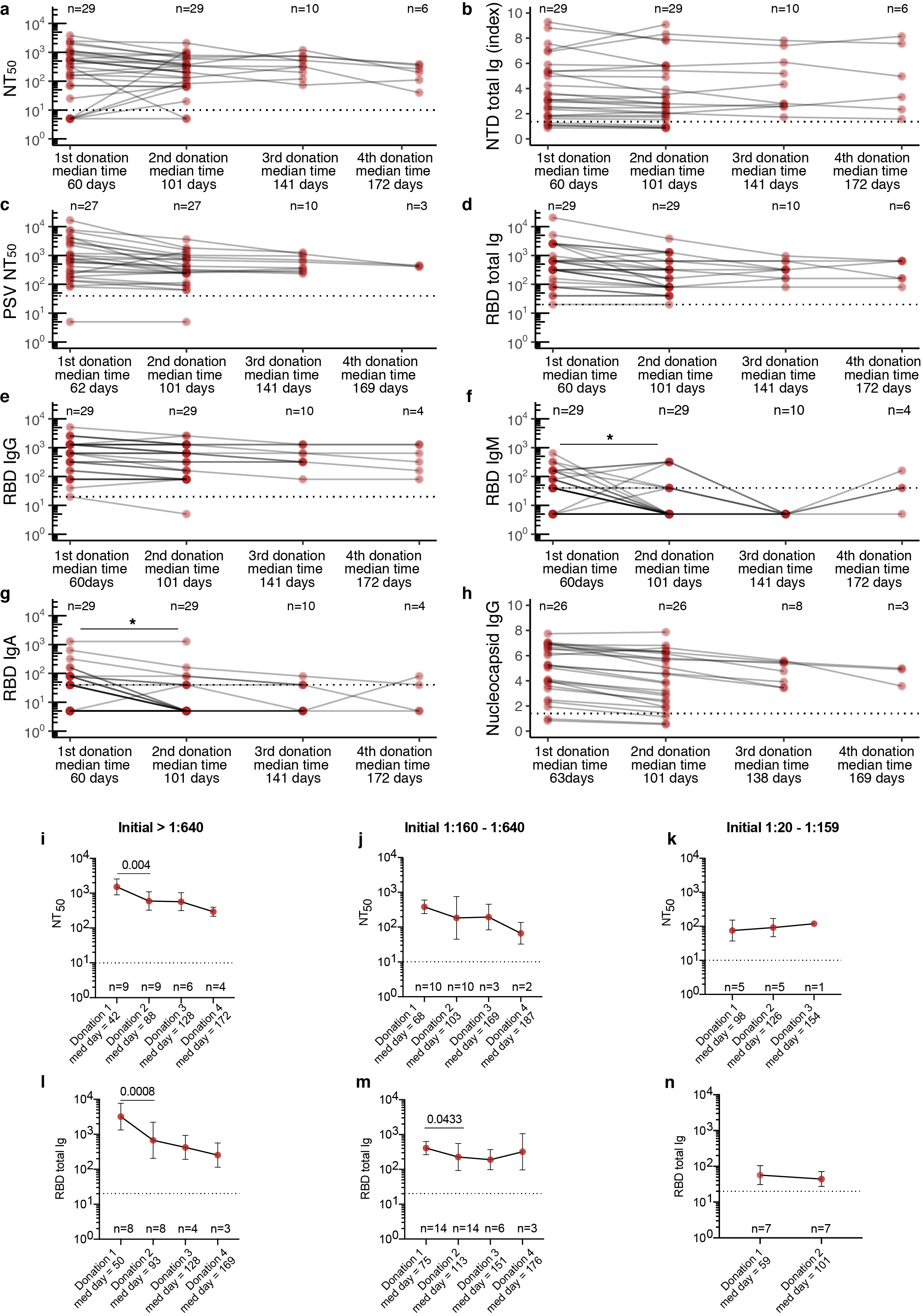

Supplement: FIG S3 [file msphere.00275-21-sf003.tif]

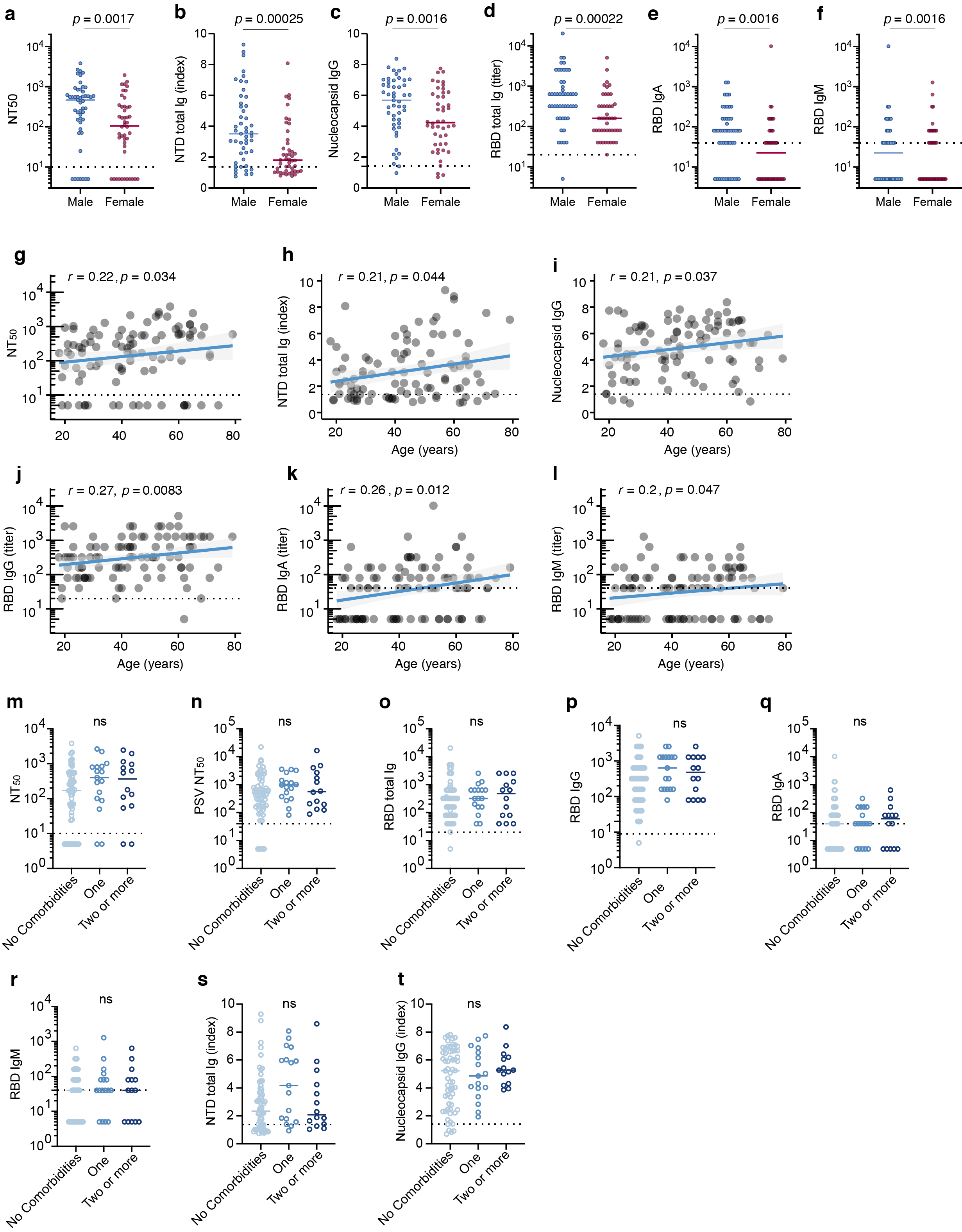

Supplement: FIG S4 [file msphere.00275-21-sf004.tif]

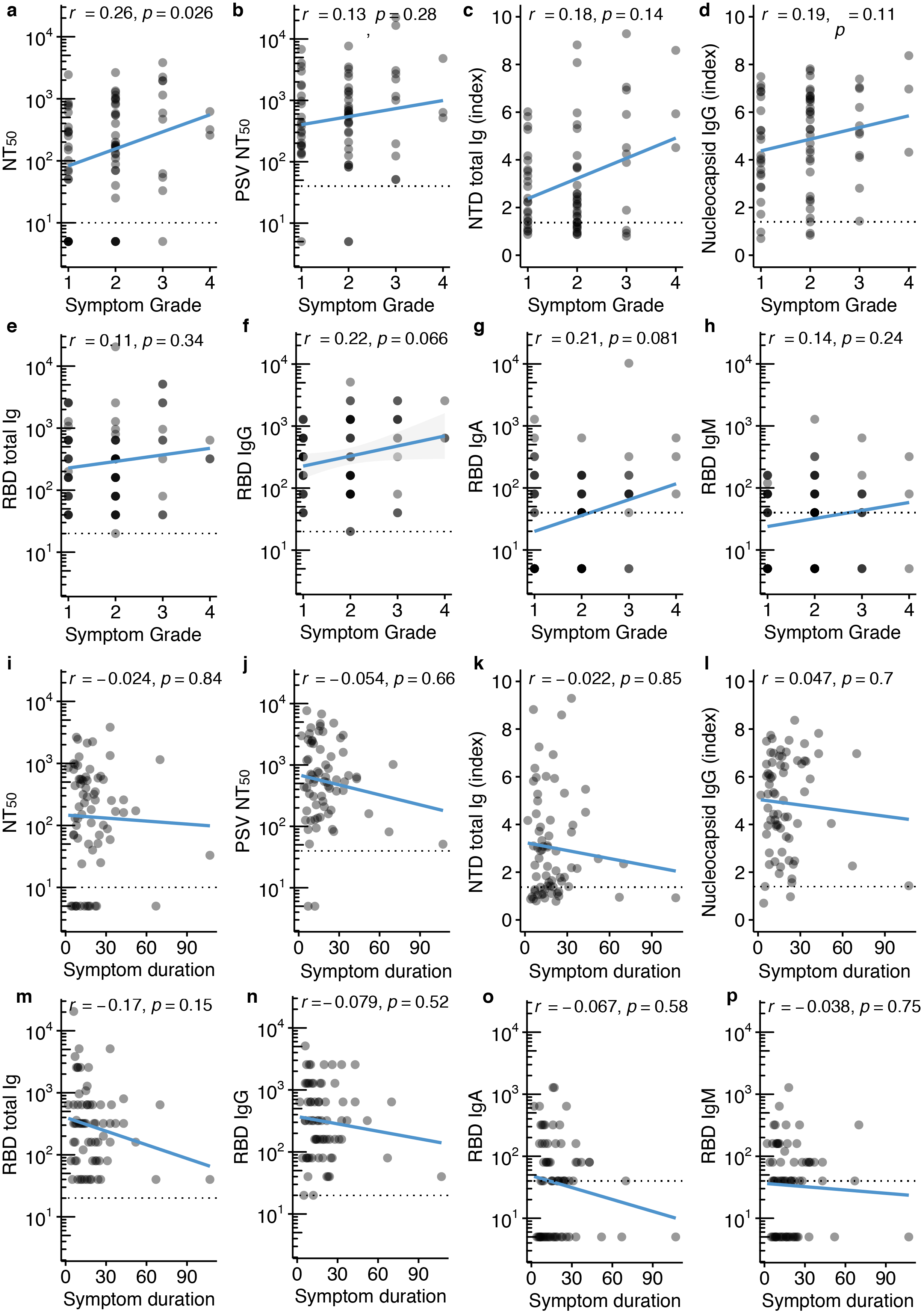

Supplement: FIG S5 [file msphere.00275-21-sf005.tif]

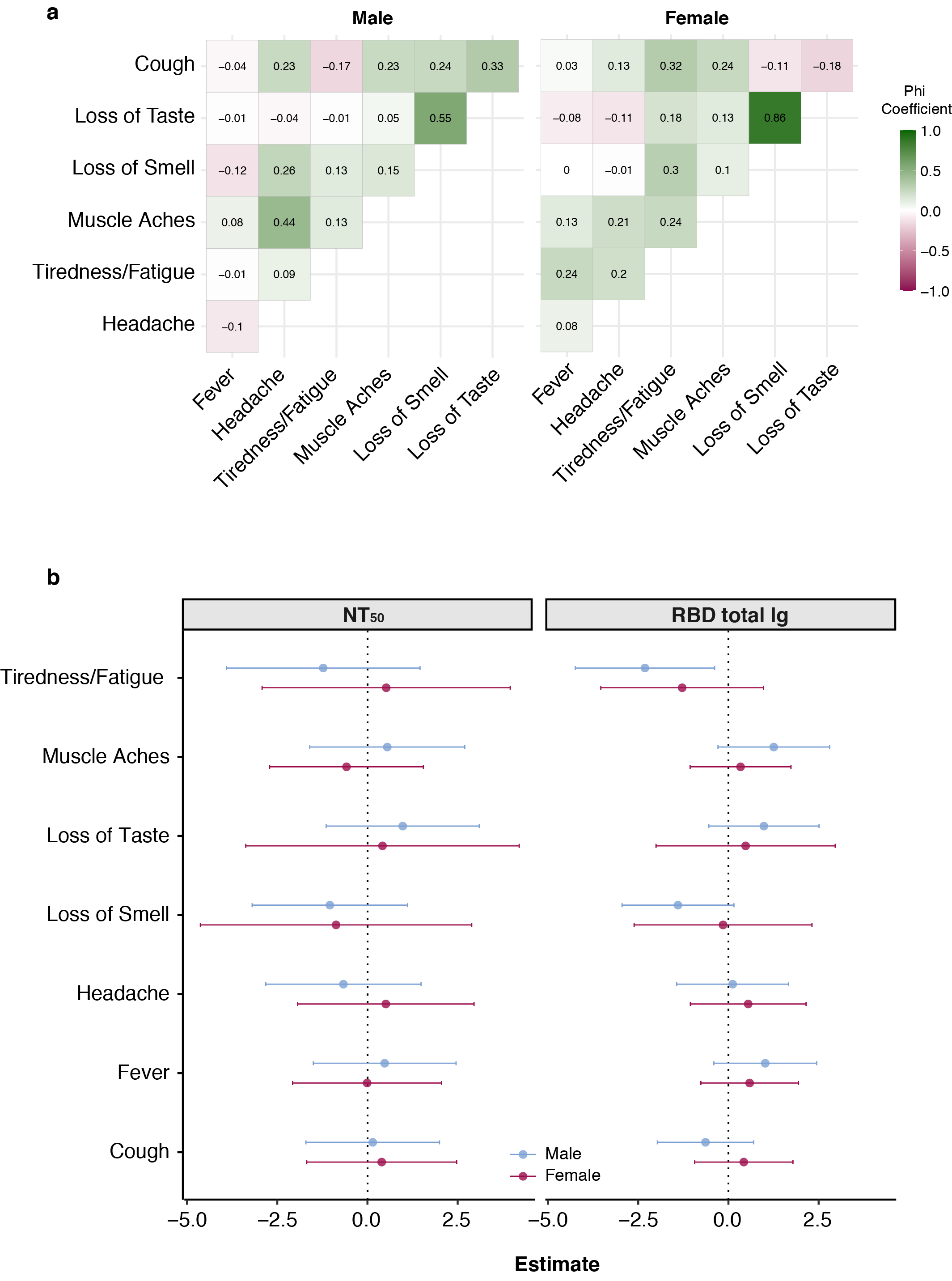

Supplement: FIG S6 [file msphere.00275-21-sf006.tif]

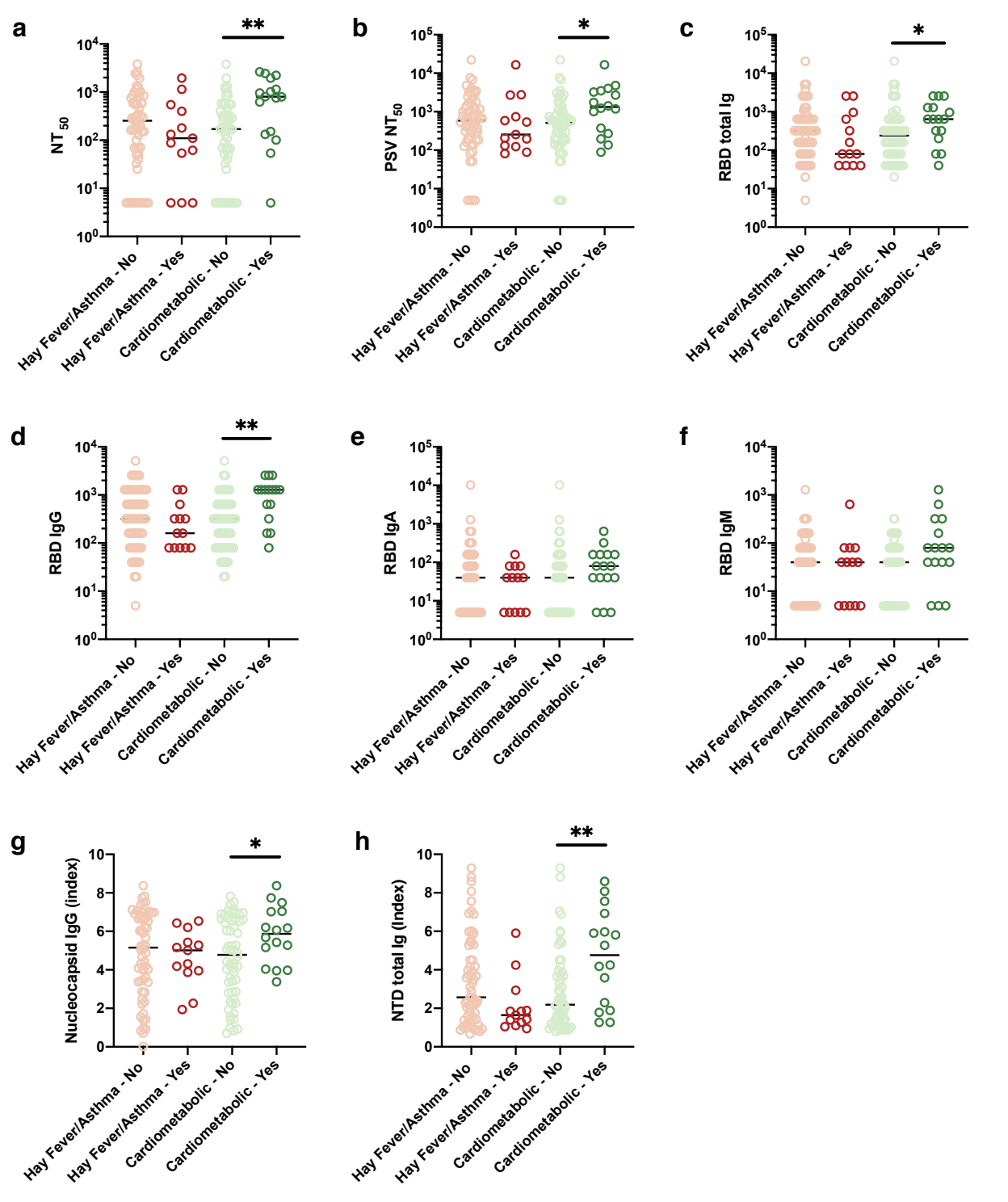

Supplement: FIG S7 [file msphere.00275-21-sf007.pdf]

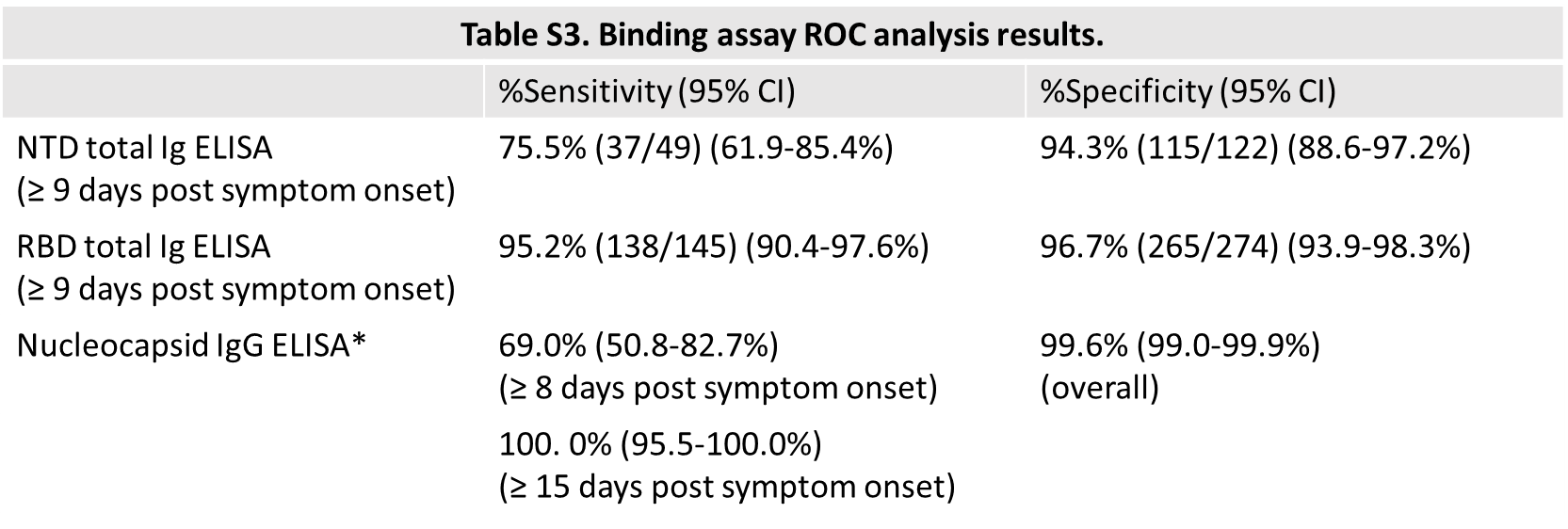


*Nucleocapsid data per manufacturer.

Supplement: TABLE S3 [file msphere.00275-21-st003.docx]
