# Supplementary material for: Sex Disparities and Neutralizing-Antibody Durability to SARS-CoV-2 Infection in Convalescent Individuals
Source: mSphere. 2021 Aug 25;6(4):e00275-21. doi: 10.1128/mSphere.00275-21 (PMC8386415; doi:10.1128/mSphere.00275-21)
Supplement: Supplementary file 2 [file msphere.00275-21-st001.docx]

Table S1. Donor SARS-CoV-2 diagnostic and symptom data.

ID#	Did the participant experience any symptoms?	Overall Symptom Grade (1-4)	Symptom Duration (Days)	Supplemental Oxygen (Y/N)	Positive Test Type (RT-PCR or Ab)	Diagnostic Test Location	Time from diagnostic test to donation
45	Yes	1	14	No	Ab	Site 1	22
57	Yes	1	16	No	Ab	Site 1	21
64	Yes	1	28	No	Ab	Site 1	34
66	Yes	1	16	No	Ab	Other Lab	27
69	Yes	1	6	No	Ab	Other Site	71
84	Yes	1	0	No	Ab	Site 1	19
3	Yes	1	15	No	RT-PCR	Site 1	33
12	Yes	1	not available	No	RT-PCR	Site 1	32
13	Yes	1	not available	No	RT-PCR	Site 1	33
16	Yes	1	9	No	RT-PCR	Site 1	38
27	Yes	1	17	No	RT-PCR	Site 1	40
30	Yes	1	not available	No	RT-PCR	Other site	45
32	Yes	1	not available	No	RT-PCR	Site 1	52
35	Yes	1	23	No	RT-PCR	Lab 1	59
40	Yes	1	not available	No	RT-PCR	Site 1	55
47	Yes	1	4	No	RT-PCR	Site 1	63
53	Yes	1	18	No	RT-PCR	Lab 1	77
76	Yes	1	6	No	RT-PCR	Site 1	43
80	Yes	1	34	No	RT-PCR	Lab 1	36
81	Yes	1	17	No	RT-PCR	Site 1	49
85	Yes	1	22	No	RT-PCR	Site 1	36
91	Yes	1	12	No	RT-PCR	Lab 1	30
98	Yes	1	2	No	RT-PCR	Lab 1	28
101	Yes	1	24	No	RT-PCR	Site 1	23
22	Yes	2	20	No	Both[†]	Site 2	3
43	Yes	2	33	No	Ab	Site 2	14
55	Yes	2	22	No	Ab	Site 1	21
56	Yes	2	5	No	Ab	Site 1	21
62	Yes	2	8	No	Ab	Site 1	20
67	Yes	2	not available	No	Ab	Lab 1	36
70	Yes	2	10	No	Ab	Lab 1	29
73	Yes	2	26	No	Ab	Site 1	23
88	Yes	2	25	No	Ab	Other site	13
100	Yes	2	52	No	Ab	Site 1	29
1	Yes	2	6	No	RT-PCR	Site 1	25
7	Yes	2	not available	No	RT-PCR	Lab 1	39
8	Yes	2	18	No	RT-PCR	Lab 1	45
14	Yes	2	9	No	RT-PCR	Lab 2	39
17	Yes	2	70	No	RT-PCR	Site 1	42
19	Yes	2	not available	No	RT-PCR	Site 1	40
21	Yes	2	31	No	RT-PCR	Site 1	51
23	Yes	2	19	No	RT-PCR	Site 1	44
34	Yes	2	19	No	RT-PCR	Lab 1	59
37	Yes	2	37	No	RT-PCR	Lab 1	49
39	Yes	2	7	No	RT-PCR	Site 1	41
44	Yes	2	11	No	RT-PCR	Lab 1	71
50	Yes	2	7	No	RT-PCR	Lab 1	51
58	Yes	2	28	No	RT-PCR	Site 1	65
68	Yes	2	15	No	RT-PCR	Site 1	84
74	Yes	2	not available	No	RT-PCR	Site 1	68
75	Yes	2	67	No	RT-PCR	Site 1	73
79	Yes	2	24	No	RT-PCR	Lab 1	36
87	Yes	2	7	No	RT-PCR	Other site	48
89	Yes	2	12	No	RT-PCR	Site 1	43
93	Yes	2	43	No	RT-PCR	Site 1	40
95	Yes	2	6	No	RT-PCR	Site 1	40
99	Yes	2	not available	No	RT-PCR	Site 1	44
10	Yes	3	9	No	RT-PCR	Site 1	37
29	Yes	3	8	No	RT-PCR	Lab 1	57
31	Yes	3	not available	No	RT-PCR	Other site	49
33	Yes	3	33	No	RT-PCR	Lab 2	54
36	Yes	3	11	Yes	RT-PCR	Lab 1	44
41	Yes	3	20	No	RT-PCR	Lab 1	71
42	Yes	3	not available	Yes	RT-PCR	Site 1	65
71	Yes	3	not available	No	RT-PCR	Site 1	68
77	Yes	3	not available	No	RT-PCR	Lab 2	95
82	Yes	3	not available	Yes	RT-PCR	Site 1	47
96	Yes	3	107	No	RT-PCR	Site 1	119
24	Yes	4	26	Yes[*]	RT-PCR	Site 1	46
72	Yes	4	43	Yes	RT-PCR	Site 1	66
97	Yes	4	21	No	RT-PCR	Site 1	110
49	Yes	not available	8	not available	Ab	Lab 1	21
59	No	not available	not available	No	Ab	Other Lab	43
60	No	not available	not available	No	Ab	Site 1	27
63	No	not available	not available	No	Ab	Site 1	12
78	No	not available	not available	No	Ab	Site 1	27
83	No	not available	not available	No	Ab	Site 1	30
86	No	not available	not available	No	Ab	Site 1	48
2	not available	not available	not available	not available	RT-PCR	Site 1	38
4	Yes	not available	8	not available	RT-PCR	Site 1	32
5	Yes	not available	13	not available	RT-PCR	Lab 2	43
6	Yes	not available	13	No	RT-PCR	Lab 2	38
9	Yes	not available	11	not available	RT-PCR	Other site	40
11	Yes	not available	16	not available	RT-PCR	Site 1	36
15	Yes	not available	14	not available	RT-PCR	Other lab	46
18	Yes	not available	9	not available	RT-PCR	Site 1	37
20	Yes	not available	12	not available	RT-PCR	Site 1	40
25	Yes	not available	not available	Yes[*]	RT-PCR	not available	60
26	Yes	not available	not available	not available	RT-PCR	Site 1	43
28	Yes	not available	9	not available	RT-PCR	Site 1	39
38	Yes	not available	not available	not available	RT-PCR	Site 1	45
46	Yes	not available	33	not available	RT-PCR	Site 1	46
48	Yes	not available	not available	not available	RT-PCR	Site 1	61
51	not available	not available	not available	not available	RT-PCR	Site 1	57
52	Yes	not available	7	No	RT-PCR	Site 1	40
54	Yes	not available	10	No	RT-PCR	Lab 1	30
61	Yes	not available	22	No	RT-PCR	Site 1	51
65	Yes	not available	16	No	RT-PCR	Other site	69
90	not available	not available	not available	not available	RT-PCR	Other Lab	62
92	No	not available	not available	No	RT-PCR	Other site	29
94	No	not available	not available	No	RT-PCR	Other site	21
