# Supplementary material for: Sex Disparities and Neutralizing-Antibody Durability to SARS-CoV-2 Infection in Convalescent Individuals
Source: mSphere. 2021 Aug 25;6(4):e00275-21. doi: 10.1128/mSphere.00275-21 (PMC8386415; doi:10.1128/mSphere.00275-21)
Supplement: Supplementary file 3 [file msphere.00275-21-st002.docx]

Table S2. Donor Comorbidities (n=34) and COVID-19 Symptoms			
Hay fever	12		
High blood pressure	10		
Asthma	7		
Cancer (ever)	7		
Diabetes	6		
Obesity	4		
Heart or cardiovascular disease	3		
Inflammatory bowel disease	3		
Blood disorder (like Sickle Cell)	1		
Asplenia or hyposplenism	1		
Liver disease	1		
Cystic fibrosis	0		
Kidney disease	0		
Lupus, systemic lupus erythematosus	0		
Multiple sclerosis	0		
**COVID-19 Symptoms**	**Yes, n (%)**	**No, n (%)**	**Total n**
Symptomatic	93 (93)	8 (7)	101
Tiredness (Fatigue)	80 (89)	10 (11)	90
Headache	69 (77)	21 (23)	90
Fever	67 (74)	23 (26)	90
Muscle Aches	66 (73)	24 (27)	90
Loss of Appetite	56 (62)	34 (38)	90
Loss of Taste	55 (61)	35 (39)	90
Loss of Smell	54 (60)	36 (40)	90
Cough	54 (60)	36 (40)	90
Shortness of Breath	44 (49)	45 (51)	89
Chills/Shaking Chills	34 (53)	30 (47)	64
Sore Throat	34 (38)	56 (62)	90
Diarrhea	31 (34)	59 (66)	90
Nasal Congestion	27 (30)	63 (70)	90
Chest Pain	25 (28)	65 (72)	90
Runny Nose	21 (24)	68 (76)	89
Nausea	19 (21)	71 (79)	90
Other Symptoms	15 (17)	75 (83)	90
Red, Itchy or Irritated Eyes	14 (16)	76 (84)	90
Sneezing	10 (13)	65 (87)	75
Vomiting	9 (10)	81 (90)	90
New Joint Pain or Swelling	8 (9)	82 (91)	90
Rash	4 (4)	86 (96)	90
New Numbness or Tingling	3 (3)	87 (97)	90
New Hearing Problems	2 (2)	88 (98)	90
